# Autoantibodies and neuropsychiatric manifestations in childhood-onset systemic lupus erythematosus

**DOI:** 10.1186/1546-0096-12-S1-P114

**Published:** 2014-09-17

**Authors:** Mariana Postal, Aline Lapa, Nailu Sinicato, Roberto Marini, Simone Appenzeller

**Affiliations:** 1 Medicine, State University of Campinas, Campinas, Brazil; 2 Pediatrics, State University of Campinas, Campinas, Brazil

## Introduction

Neuropsychiatric manifestations are considered to be a serious complication in childhood-onset systemic lupus erythematosus (cSLE). The pathogenesis of neuropsychiatric manifestations has been attributed to autoantibody-mediated neural dysfunction.

## Objectives

To investigate the prevalence and associations of neuropsychiatric manifestations with antiribosomal P protein antibodies, S100β, subunit of high molecular weight neurofilament (NF-H), antiphospholipid [anticardiolipin (aCL) and lupus anticoagulant (LA)], anti-dsDNA and anti-Smith.

## Methods

We included consecutive cSLE followed at the pediatric rheumatology unit of the State University of Campinas. Neurological manifestations were analyzed according to the ACR classification criteria. Cognitive evaluation was performed in all participants using Wechsler Intelligence Scale for children (WISC-III) and Wechsler Intelligence Scale for adults (WAIS), according to age and validated in Portuguese. Mood disorders were determined through Becks Depression and Anxiety Inventory in all participants. SLE patients were further assessed for clinical and laboratory SLE manifestations, disease activity [SLE Disease Activity Index (SLEDAI)], damage [Systemic Lupus International Collaborating Clinics/American College of Rheumatology Damage Index (SDI)] and current drug exposures. Antiribosomal P protein antibodies, S100β and NF-H were tested by ELISA using commercial kits. The levels of dsDNA antibodies were determined by indirect immunofluorescence using *Crithidia*as a substrate and were considered positive if they were higher than 110. The levels of precipitating antibodies to extractable nuclear antigens (ENA), including Sm, were detected using a standardized enzyme-linked immunosorbent assay (ELISA) method and were considered positive if higher than 180. The levels of IgG and IgM anticardiolipin antibodies (aCL) were measured by ELISA. Lupus anticoagulant (LA) activity was detected by coagulation assays in platelet-free plasma obtained by double centrifugation following the recommendations of the Scientific and Standardization Committee of the International Society of Thrombosis and Homeostasis subcommittee on LA Antiphospholipid (aCL and LA), anti-dsDNA and anti-Smith were obtained of the medical charts. Data were compared by non-parametric tests.

## Results

We included 77 cSLE patients (69 women; mean age 17.64±4.64 years). The mean disease duration was 4.35±3.9 years . At time of study entry, 33 (42.85%) cSLE patients had active disease (mean SLEDAI scores 3.58±3.97; range 0-14). Eighteen (23.37%) cSLE had cumulative damage (mean SDI scores 0.35±0.67; range 1-3). We observed neuropsychiatric manifestations in 49 (63.63%) cSLE. The most frequent manifestations observed in our cohort were cognitive impairment (46.93%), depression (36.73%) and seizure (24.48%). Mean NF-H protein levels were 101.80±89.40 pg/mL and mean serum S100β levels were 148.98 ± 102.73 pg / mL in cSLE. antiP were positive in 13 (16.8%), aCL in 15 (19.5%), LA in 29 (37.7%), dsDNA in 31 (40.3%) and anti-Sm in 11 (14.3%) cSLE patients. We did not observe an association between the presence of neuropsychiatric manifestations and any of the tested autoantibodies.

## Conclusion

Although frequently observed in cSLE antiribosomal P protein antibodies, aCL and LA, anti-dsDNA and anti-Smith, S100B and NF-H are not good biomarkers for neuropsychiatric manifestations.

## Disclosure of interest

M. Postal: None declared., A. Lapa: None declared., N. Sinicato: None declared., R. Marini: None declared., S. Appenzeller Grant / Research Support from: FAPESP (2008/02917-0, 2011/03788-2, 2013/09480-5); CNPQ (300447/2009-4 and 471343/2011-0; 302205/2012-8; 473328/2013-5).

